# Patients’ experience of Chinese Medicine Primary Care Services: Implications on Improving Coordination and Continuity of Care

**DOI:** 10.1038/srep18853

**Published:** 2015-12-21

**Authors:** Vincent CH Chung, Benjamin HK Yip, Sian M Griffiths, Ellen LM Yu, Siya Liu, Robin ST Ho, Xinyin Wu, Albert WN Leung, Regina WS Sit, Justin CY Wu, Samuel YS Wong

**Affiliations:** 1 JC School of Public Health and Primary Care, The Chinese University of Hong Kong, Hong Kong, Hong Kong SAR, China; 2 Hong Kong Institute of Integrative Medicine, The Chinese University of Hong Kong, Hong Kong, Hong Kong SAR, China; 3 School of Chinese Medicine, The Chinese University of Hong Kong, Hong Kong, Hong Kong SAR, China

## Abstract

Chinese medicine (CM) is major form of traditional and complementary medicine used by Chinese populations. Evaluation on patients’ experience on CM service is essential for improving service quality. This cross sectional study aims (i) to assess how CM clinics with different administrative model differ in terms of quality from patients’ perspective; and (ii) to investigate how quality varies with patients’ demographic and health characteristics. Five hundred and sixteen patients were sampled from charity and semi-public CM clinics in Hong Kong, and were invited to assess their experience using the Primary Care Assessment Tool (PCAT). Results indicated that overall mean PCAT scoring is satisfactory, achieving 70.7% (91.26/129) of total score. Ratings were lower in areas of “coordination of patient information”, “continuity of care”, and “range of service provided”. Impact of administrative models, including involvement of tax-funded healthcare system and outreach delivery, were minimal after adjusting for patient characteristics. Demographic and health characteristics of patients did not contribute to substantial variations in scoring. To improve patient experience, policy makers should consider strengthening care coordination, continuity and comprehensiveness in CM primary care services. Sharing of electronic records and establishing referral system are potential solutions for linking CM and conventional healthcare services.

In response to patients’ choice^1^ and emerging clinical evidence on traditional and complementary medicine (T & CM)^2^,^3^,^4^, the World Health Organization (WHO) is advocating the integration of T&CM into national health system in its *Traditional Medicine Strategy 2014-2023*^5^. In many national health systems, conventional medicine is the dominant type of primary care practice, and patients often obtain T&CM service from the private sector simultaneously, leading to fragmentation of care^6^. The provision of both conventional and T&CM within the same health services delivery infrastructure can ensure orchestrated delivery of both services^7^. Integrative delivery echoes suggestion from the *2008 World Health Report Primary Health Care – Now More than Ever,* in which health systems are encouraged to consolidate modalities of healthcare horizontally across the community - including both formal and informal providers in public and private sectors^8^.

In Hong Kong, Chinese medicine (CM) is the major form of T&CM, and its use is popular among chronic disease patients who are already receiving conventional care^9^. After the reunification of Hong Kong with China in 1997, the government has implemented development plans for CM as a fulfillment to constitutional mandate. Formal regulation on CM practitioners and Chinese herbal medicines are implemented, and CM practitioners are positioned as a parallel profession to conventional medical doctor. Nevertheless, bridge building and interprofessional education between the two professions have been limited^10^. On the service provision side, initiatives in establishing CM service beyond the private sector has started a decade ago, but it is largely positioned outside the tax funded healthcare system^11^. The public healthcare system is managed by the Hospital Authority, which provides conventional medical services at various levels. On the other hand, non-governmental organizations (NGOs) are the main providers of charity and semi-public CM services^12^.

## Charity and semi-public TCM services in Hong Kong

For non-profit charity service, NGOs manage two types of CM delivery models: (i) outpatient clinics (NGOs clinics) and (ii) mobile “clinics” which provide outreach CM services via fully equipped vans (mobile clinics). The NGOs are also actively involved in the provision of semi-public CM services by managing Clinical Centers for Teaching & Research in Chinese Medicine (CCTRCM). CCTRCM are established under the tripartite collaboration between NGOs, universities providing tertiary CM education, as well as the Hospital Authority. CCTRCM distinguish themselves from NGOs and mobile clinics as they have (i) formal linkages with the tax funded healthcare system^13^, and (ii) consultation fee waivers mechanism for patients receiving social security^14^ . Regardless of their management structure, all clinics provide three types of CM services: herbal medicine, acupuncture and massage therapy.

## Aim of this study

Internationally, T&CM service provision within charity and semi-public setting is a relatively new endeavor in health systems with a dominant conventional care sector. Providing quality T&CM care is challenging especially in maintaining coordination and continuity of care across T&CM and conventional care sectors for chronic disease patients, especially those with multiple morbidities^15^. Little is known on how these new initiatives in Hong Kong are equipped to manage these challenges, and also on how different administrative and service delivery models may affect quality of care. This study aims to assess patient’s experience of primary care provided by charity and semi-public CM clinics. The objectives were (i) to assess how CCTRCM, NGOs and mobile clinics differ in terms of quality from patients’ perspective; and (ii) to investigate how quality varies with patients’ demographic and health characteristics.

## Methods

### Sampling and Data Collection

This study was conducted in charity and semi-public CM clinics managed by a NGO in Hong Kong. Participants were selected using stratified sampling in three types of CM clinics mentioned above. These include five NGO clinics, eighteen mobile clinics and two CCTRCM. Following the sample size requirement of 20 subjects per independent variable in conducting multiple linear regression analysis^16^, we estimated that the required sample size would be 500. To ensure representative recruitment of patients, we sampled 167 patients from each type of clinic. Patients who were: (i) aged 18 or above, (ii) able to provide written informed consent, (iii) able to read and write Chinese without assistance were invited to participate. Specifically, we approached thirty-four patients from each of the 5 NGO clinics, ten patients from each of the 18 mobile clinics, and eighty-three patients from each of 2 CCTRCM. In each clinic, all eligible service users invited to participate in a face to face interview immediately after consultation. We invited consecutive patients who attended the clinics until the required sample size was reached. Written informed consent was obtained from patients prior to the interview. The research protocol was approved by the Joint Chinese University of Hong Kong – New Territories East Cluster Clinical Research Ethics Committee (Reference number: CRE-2012.113). The methods were carried out in accordance with the guideline approved by the Committee.

### Questionnaire Design

The questionnaire used in the interview consisted of two parts. The first part aimed to collect data on respondents’ demographic and health-related characteristics, including their gender, age, employment status, education level, marital status, housing type, payment status, reason for consultation, duration of waiting time, and duration of consultation. We also queried on patients’ self-perceived health status using the validated question of: “In general, how would you rate your health?” [in Cantonese] with response options of excellent, very good, good, fair, or poor^17^.

In the second part, we assessed patients’ experience in CM service using by using the validated Primary Care Assessment Tool (PCAT) Adult edition short version instrument. The Chinese version used in this study was found to have good reliability and validity^18^.

PCAT assesses patients’ experience in the following domains, which are to be key attributes of quality primary care services^19^: *first contact accessibility and utilization, continuity of care, coordination of services and information system, comprehensiveness of services availability and provision, family centeredness, community orientation, and cultural competence*. Question items in each domain were rated on a 1 to 4 Likert scale. If a “not sure/cannot remember” option was chosen, scorings were imputed using method described in the statistical analysis section. Total score for each domain was calculated by summing (with reverse coding whenever appropriate) the values for all items within each domain. The overall PCAT score was derived by summing sores from each domain.

### Statistical Analysis

Pearson’s Chi-square test was used to compare the socio-demographic and health related characteristics of patients in different healthcare settings. Variables with significant difference (p-value <0.15) were treated as potential confounder in the multiple regression analysis. There was 5.3% of missing values and 11.8% of “Don’t Know/Can’t Remember” options in the PCAT data, and these were which imputed by the expectation-maximization (EM) algorithm with single imputation^20^. Subsequently, the domain and total scores of PCAT among different types of CM clinics were compared using multiple linear regression by adjusting patients’ demographic and health characteristics (independent variables included patients’ gender, age, education level, employment status, marital status, types of housing, self-perceived health status, Chinese Medicine specialty visited, reason for consultation, and usage of conventional specialist services in public sector). Multiple linear regression analysis using full information maximum likelihood was performed to assess the association between PCAT total score and patients’ socio-demographic and health related characteristics^20^. Same regression model were used as sub-analyses for each PCAT domain score. Almost all patients from NGO or mobile clinics need to pay, but 30.7% patients sampled in the CCTRCM had their consultation fee waived. We conducted sensitivity analysis to study the potential influence of payment status on PCAT score by using CCTRCM data only. Statistical significance was set at p < 0.05. All analyses were performed using R version 2.15.2^21^, in which the package OpenMx was used for multiple linear regression using full information maximum likelihood^22^,^23^.

## Results

### Characteristics of respondents

Table 1 presents the socio-demographic and health characteristics of 516 participants sampled from NGO clinics, mobile clinics and CCTRCM. The distribution of gender, age, education level, employment status, marital status, TCM specialty visited, reason for consultation, consultation payment and specialist visited experience differed significantly among three types of clinics, except for type of housing and self-perceived health status.

### PCAT scorings

Results on PCAT score are reported in Table 2. Overall analysis of data from all three types of clinics showed that the PCAT total score is satisfactory, with a mean rating of 91.26 out of 129. In other words, rating achieved 70.7% of maximum possible score. Nevertheless, mean scorings in the domains of *Continuity of Care (8.64 out of 16), Coordination (Information System) (6.79 out of 12), Comprehensiveness (Service provided) (12.43 out of 16)* and *Cultural Competence (6.11 out of 12)* were lower. In these domains, ratings were below 60% of highest possible scores.

### Comparison of PCAT score across CCTRCM, NGO and mobile clinics

Multiple linear regression analysis showed that, after adjusting for socio-demographic and health characteristics, all other PCAT domain scores and total score for NGO and mobile clinics differed significantly from that of CCTRCM clinics, except the first two domains in *First Contact* (Table 2). For *Continuity of Care*, CCTRCM had the highest mean score, while NGO clinics had the lowest mean score. Also, CCTRCM had higher score than that of the NGO and mobile clinics in the domain of *Cultural Competence*. However, when compared to CCTRCM users, both NGO and mobile clinic patients had slightly better experiences in the following domains: *Coordination of Service, Coordination (information system), Comprehensiveness of Service Available and Provided, Family Centeredness and Community Orientation*. Overall, NGO and mobile clinics scored slightly higher in total PCAT scores when compared to CCTRCM. The mean differences in PCAT total scores between CCTRCM and NGO / mobile clinics are less than 6 out of a total of 129, indicating that there is no substantial difference in patient experiences among the clinic types.

### Association between PCAT scoring, clinic type and patients’ characteristics: Multiple linear regression analysis

Table 3 presents results from multiple linear regression analysis on total PCAT score, with clinic type as well as patients’ socio-economic and health characteristics as independent variables. Our results indicated that patients from NGO or mobile clinics reported a higher overall PCAT score when compared to those using CCTRCM. Retired patients rated a higher score than those who were employed. Magnitudes of associations described above were weak, as all the standardized β (adjusted) values are below 0.20. No other characteristics were found to be significantly associated with PCAT total score.

### Association between PCAT scoring and patients’ characteristics in CCTRCM: Multiple linear regression analysis

Table 4 presents results from multiple linear regression analysis performed on data from CCTRCM, with payment of consultation fee as an extra independent variable. The results indicated that patients with primary education or below tended to rate their overall score lower than those received tertiary education. Unemployed patients rated a higher score than those who were employed. Compare to patients who consulted an herbalist, those who received acupuncture treatment reported a significantly lower score. Magnitudes of associations described above were weak, with standardized β (adjusted) values vary from 0.22 to 0.24.

## Discussion

This is one of the first studies that evaluate patients’ experience of primary care provided by charity and semi-public CM clinics. While previous studies have reported poorer quality of conventional care received by Hong Kong patients with lower socioeconomic background^16^, in this CM focused study PCAT domain scores were largely similar across patients with different demographic characteristics. While the overall patient experience is satisfactory, ratings are lower in the three areas of *“coordination of patient information”, “continuity of care”, and “range of service provided”*. Impact of administrative models on the results, including involvement of tax-funded healthcare system and outreach delivery, are minimal after adjusting for demographic and health characteristics of patients. When compared to a previous PCAT study that evaluated patients’ experiences in conventional public and private medical services in Hong Kong^18^, CM clinics fared worse than both public and private conventional care service in coordinating of patient information. For ensuring continuity of care, CM clinics were on par with public conventional clinics, but not with private primary care service. It can be speculated that lower PCAT score in CM clinics could be attributed to the lack of channels for communication and information sharing between the CM and conventional care sectors.

### The need of improving information coordination and continuity across CM and conventional care

The PCAT domain of *Coordination (information system)* assesses three aspects: (i) readiness of patient record prior to consultation; (ii) patients’ right to examine their own record; and (iii) whether patients can own a copy of their record and bring it to the consultation. As the use of electronic health record systems is widespread in all three types of CM clinics, patients’ case notes are often ready at the consultation. Also, patients are allowed to read their record if requested. The main reason for lower score in this domain is patients’ difficulties in bringing their conventional care record to CM consultations. There is no formal referral system between CM and both public and private biomedical services^24^ except for certain selected diseases^13^. Communication across CM and conventional care providers using referral letters is uncommon. Even in CCTRCM where the Hospital Authority is involved, seamless electronic health record sharing between CM and tax-funded biomedical care are not in place. Responsibility of coordinating information flow across CM and conventional clinicians falls solely on the patients. In order to obtain their own CM and conventional health records, separate administrative fees must be paid in CM and conventional care settings respectively. The lack of communication channel with conventional health services, together with barriers for patients in obtaining their own records, has led to poor coordination of patient information in CM settings. As the government is promoting a territory wide sharing of electronic record system^25^, coordination of information across the two sectors may improve in the future. Unfortunately, there is no consensus between CM and conventional medicine stakeholders on whether patient record should be shared across disciplines^26^. Further policy research is needed for formulating informational sharing protocol that would be acceptable to both patients and professionals.

The PCAT domain of *Continuity of Care* assesses three aspects: (i) whether patients can consult the same CM clinician in every episode; (ii) whether patients can ask the same CM clinician questions with regards to their condition; and (iii) whether the CM clinician knows the patient well. It appears that private conventional care is performing better in these aspects. A common problem for both CM and public conventional care is that patients are often unable to see the same clinician, and contacting clinician who manages their last consultation is usually not facilitated. This hinders continuity of care within CM or conventional care, not to mention fragmentation of care across two types of care^27^. The combined impact of poor information coordination and lack of continuity could pose risk to patients, especially when Chinese herbs and conventional drugs are prescribed by two clinicians without acknowledging each other^28^. For example, common Chinese herbs like *Angelica sinensis* or *Salvia miltiorrhiza* can potentiate the effect of warfarin, leading to excessive bleeding tendency among patients^29^. Potential threats of adverse herb-drug interaction demonstrate the need of improving coordination and continuity across CM and biomedical care at policy level^30^.

Beyond reducing the risk of herb-drug interaction, streamlining both services can also improve connectivity and trust between CM and biomedical clinicians. This is particularly important for policymakers who are interested in developing integrative model of care, which is gaining popularity internationally^7^. Successful collaboration between T&CM and conventional clinicians depends on timely information exchange and efficient referral platform. Shared electronic health record is a preferred channel for communication^31^,^32^,^33^, and face-to-face case conferences would further enhance the quality of coordination^34^. Co-location of CM and conventional clinicians is a potential strategy that can facilitate more frequent communications^34^,^35^,^36^. Nevertheless, these benefits cannot be reaped unless sufficient interprofessional education is provided for both types of clinicians^37^, and the potential legal barriers of patient record sharing across CM and biomedical clinicians are cleared^38^.

### Building capacity in providing wider range of services: Resource implications for managing complex health needs

Our results indicated that scoring on the “*service provided*” domain is low, and similar pattern is found in both public and private conventional care. Low level of advice provided on diet, sleep, exercise, smoking cessation, medication use, home safety and family conflict provided may be attributed to short consultation time available in Hong Kong, which lasts only 5–7 minutes per episode^39^. Current evidence suggests that primary care based health promotion can provide some benefits to patients^40^,^42^. To relieve burden from clinicians, it is recommended that these tasks can be delegated to other primary care team members^41^. The possibilities of using a coordinated, team based approach for addressing multiple health promotion needs in primary care setting should be explored, in both CM and biomedical care settings^43^.

### Strengths and Weaknesses of This Study

One of the strengths of this study is that we reduced selection bias by using a stratified sampling strategy. This enabled us to recruit a representative sample of CM users from all three types of clinics. This study compared patients’ experience in CCTRCM, NGO and mobile clinics, of which each has different administrative and delivery model, and we aimed at investigating the overall impact of such model. Involvement of the tax funded healthcare system and fee waiver mechanism for patients receiving social security are two distinctive features of CCTRCM. Our primary focus was not to investigate how each feature may influence patients’ experience. That said, to clarify that our findings, we performed a sensitivity analysis and showed that payment has no significant impact on patient experience in CCTRCM.

Meanwhile, this study has several limitations. First, attendees were asked to assess service quality within the CM clinic, and thus social desirability bias could have led to inflation of score. Second, due to privacy reason, we were unable to obtain the full attendance lists of the clinics as a sampling frame. Therefore, we have chosen a non-probabilistic, consecutive sampling approach. The response rate for CCTRCM, NGO clinics and mobile clinics were 58.8%, 58.1% and 57.6% respectively. Since the response rate for all three types of clinics are similar, we do not expect significant self-selection bias introduced by patients who have positive preference on a particular style of service.

Lastly, in the discussion section, we have attempted to compare our results with findings on conventional care quality. This comparison should be considered preliminary as we did not sample patients who used both CM and conventional healthcare services. Future research should focus on evaluating the quality of information coordination and continuity among patients who consult both types of clinicians, which will provide direct evidence on whether the postulated differences between two types of care exists. Quantitative results from such investigation can be triangulated with qualitative findings, which will provide more policy relevant insights. For example, focus groups or in-depth interviews maybe conducted for exploring patients’ experiences in navigating both types of care, of which results can be translated to practical, patients centered strategies for integrative service re-design.

## Conclusion

Quality of primary care provided by charity and semi-public CM clinics, assessed from patients’ perspective using PCAT, is satisfactory overall. However, there are rooms for improvement in the aspects of information coordination, continuity of care, and range of health promotion and preventive service provided. Such weaknesses may be attributed to these clinics’ lack of connectivity with the conventional care sector. Exclusion of CM from the tax funded healthcare system also limited its role in delivering comprehensive care for patients who wish to use both types of services. Streamlining CM and conventional care, especially by sharing of electronic health record and establishing referral system, may improve quality of care. Policy makers should also consider the enhancement of CM sectors’ capacity in providing a more comprehensive range of services. Experience from Hong Kong can serve as a pioneering example for health systems which are considering the integration of T&CM and conventional care.

## Additional Information

**How to cite this article**: Chung, V. C.H. *et al.* Patients’ experience of Chinese Medicine Primary Care Services: Implications on Improving Coordination and Continuity of Care. *Sci. Rep.* **5**, 18853; doi: 10.1038/srep18853 (2015).

## Figures and Tables

**Table 1:** Comparison of Socio-demographic and Health Related Characteristics of Respondents.

	CCTRCM (n = 166) n%	NGO Clinics (n = 170) n%	Mobile Clinics (n = 180) n%	p*	Total# (N = 516) n%	
Gender						
Male	82(49.4)	45(26.5)	45(25.0)	<0.001	172 (33.3)	
51Female	84(50.6)	124(72.9)	134(74.4)		342 (66.3)	
Age						
15–29	25(15.1)	32(18.8)	18(10.0)	0.026	75 (14.5)	
30–39	17(10.2)	35(20.6)	27(15.0)		79 (15.3)	
40–49	37(22.3)	33(19.4)	41(22.8)		111 (21.5)	
50–59	38(22.9)	31(18.2)	50(27.8)		119 (23.1)	
60–69	33(19.9)	19(11.2)	23(12.8)		75 (14.5)	
>70	15(9.0)	20(11.8)	16(8.9)		51 (9.9)	
Education level						
Primary education or below	28(16.9)	45(26.5)	46(25.6)	0.002	119 (23.1)	
Secondary education	83(50.0)	70(41.2)	101(56.1)		254 (49.2)	
Tertiary education or above	55(33.1)	54(31.8)	32(17.8)		141 (27.3)	
Employment status						
Employed	98(59.0)	97(57.1)	72(40.0)	0.003	267 (51.7)	
Unemployed	23(13.9)	18(10.6)	29(16.1)		70 (13.6)	
Retired	24(14.5)	34(20.0)	46(25.6)		104 (20.2)	
Others	14(8.4)	20(11.8)	30(16.7)		64 (12.4)	
Marital status						
Married/cohabitation	104(62.7)	102(60.0)	128(71.1)	0.023	334 (64.7)	
Single	46(27.7)	52(30.6)	29(16.1)		127 (24.6)	
Divorced/separated/widowed/others	16(9.6)	15(8.8)	22(12.2)		53 (10.3)	
Type of housing						
Private house	102(61.4)	108(63.5)	128(71.1)	0.149	338 (65.5)	
Public house/others	62(37.3)	61(35.9)	51(28.3)		174 (33.7)	
Self-perceived health status						
Excellent /very good	21(12.7)	12(7.1)	13(7.2)	0.058	46 (8.9)	
Good	52(31.3)	49(28.8)	47(26.1)		148 (28.7)	
Fair	77(46.4)	98(57.6)	94(52.2)		269 (52.1)	
Poor	16(9.6)	11(6.5)	26(14.4)		53 (10.3)	
Chinese medicine specialty visited						
Herbal medicine	129(77.7)	139(81.8)	135(75.0)		0.009 403 (78.1)	
Bone setting/massage	20(12.0)	12(7.1)	8(4.4)		40 (7.8)	
Acupuncture	17(10.2)	15(8.8)	32(17.8)		64 (12.4)	
Reason for consultation						
Episodic illness	71(42.8)	80(47.1)	63(35.0)	0.017	214 (41.5)	
Chronic condition	77(46.4)	84(49.4)	84(46.7)		245 (47.5)	
Both conditions/others	18(10.8)	6(3.5)	23(12.8)		47 (9.1)	
Need to pay for consultation fee						
No	51(30.7)	1(0.6)	4(2.2)	<0.001	56 (10.9)	
Yes	108(65.1)	167(98.2)	175(97.2)		450 (87.2)	
Usage conventional specialist services in public sector						
No	83(50.0)	53(31.2)	79(43.9)	0.001	215 (41.7)	
Yes	69(41.6)	105(61.8)	96(53.3)		270 (52.3)	

Keys: CCTRCM, Clinical Centers for Teaching & Research in Chinese Medicine; NGO, Non-Governmental Organization.

*p-value: Derived from Pearson’s Chi square test.

^#^In some categories, sum of percentages does not equal to 100% because of missing data.

**Table 2:** Comparison of Primary Care Assessment Tool Domain and Total Scores among Different Types of Chinese Medicine Clinics.

PCAT domain	Range of score	Total	CCTRCM	NGO Clinics	Mobile Clinics	MD NGO vs CCTRCMMD_adj_ (SE)^†^	MD Mobile vs CCTRCMMD_adj_ (SE)^†^
		(N = 516)	(n = 166)	(n = 170)	(n = 180)		
		Mean (SD)	Mean (SD)	Mean (SD)	Mean (SD)		
1 First contact (Utilization)	(3–12)	7.80(2.11)	7.62(1.92)	7.92(2.12)	7.84(2.25)	0.26(0.24)	0.30(0.24)
2 First contact (Accessibility)	(4–16)	11.05(2.42)	11.13(2.27)	10.84(2.08)	11.16(2.81)	−0.37(0.27)	−0.05(0.28)
3 Continuity of care	(4–16)	8.64(3.17)	10.15(2.95)	7.46(2.58)	8.36(3.32)	−2.31(0.33)^***^	−1.35(0.33)^***^
4 Coordination of services	(4–16)	11.11(2.85)	10.09(2.38)	11.93(2.66)	11.27(3.14)	1.49(0.31)^***^	0.85(0.31)^**^
5 Coordination (Information system)	(3–12)	6.79(2.12)	6.16(1.97)	7.01(1.85)	7.17(2.36)	0.85(0.24)^***^	1.01(0.24)^***^
6 Comprehensiveness (Service available)	(4–16)	11.18(3.54)	9.95(3.51)	11.80(3.04)	11.73(3.72)	1.67(0.39)^***^	1.39(0.40)^***^
7 Comprehensiveness (Service provided)	(5–25)	12.43(3.81)	10.58(2.97)	13.36(3.67)	13.26(4.03)	2.87(0.40)^***^	2.48(0.41)^***^
8 Family centeredness	(3–12)	7.32(2.08)	6.85(1.61)	7.56(2.18)	7.54(2.29)	0.76(0.23)^**^	0.77(0.24)^**^
9 Community orientation	(3–12)	8.84(2.12)	7.87(1.90)	9.29(2.00)	9.30(2.13)	1.44(0.23)^***^	1.32(0.23)^***^
10 Cultural competence	(3–12)	6.11(2.07)	7.04(1.96)	5.93(2.01)	5.41(1.90)	−1.00(0.22)^***^	−1.37(0.23)^***^
PCAT total score	(65–129)	91.26(14.91)	87.44(11.70)	93.11(13.72)	93.03(17.78)	5.66(1.67)^***^	5.34(1.69)^**^

Keys: PCAT, Primary Care Assessment Tool; CCTRCT, Clinical Centers for Teaching & Research in Chinese Medicine; MD, mean difference; NGO, Non-Governmental Organization.

^†^Adjusted mean difference and standard errors derived from multiple linear regression using full information maximum likelihood. The independent variables included patients’ gender, age, education level, employment status, marital status, types of housing, self-perceived health status, Chinese Medicine specialty visited, reason for consultation and usage of conventional specialist services in public sector.

*p<0.05; **p<0.01; ***p<0.001.

**Table 3:** Association between PCAT Total Score, Clinic Type, and Patients’ Characteristics: Multiple Linear Regression.

	Unstandardized β_adj_ (SE)^†^		Standardized β_adj_ (SE)^†^		Z	*p*
Health care setting						
CCTRCM^*^						
NGO clinic	5.66	(1.67)	0.18	(0.05)	3.394	<0.001
Mobile clinic	5.34	(1.69)	0.17	(0.05)	3.170	0.002
Gender						
Male^*^						
Female	0.32	(1.46)	0.01	(0.05)	0.220	0.826
Age						
15–29^*^						
30–39	−2.59	(2.80)	−0.06	(0.07)	−0.924	0.355
40–49	0.04	(2.91)	0.00	(0.08)	0.013	0.989
50–59	0.62	(3.04)	0.02	(0.09)	0.205	0.838
60–69	−1.95	(3.73)	−0.05	(0.09)	−0.524	0.600
>70	0.55	(4.23)	0.01	(0.09)	0.129	0.897
Type of housing						
Private housing^*^						
Public housing/others	1.74	(1.40)	0.06	(0.04)	1.245	0.213
Marital status						
Married/cohabitation^*^						
Single	0.85	(2.21)	0.03	(0.07)	0.384	0.701
Divorced/separated/widowed/others	−2.07	(2.23)	−0.04	(0.05)	−0.926	0.354
Education level						
Tertiary education or above^*^						
Primary education or below	−1.31	(2.30)	−0.04	(0.07)	−0.568	0.570
Secondary education	−2.50	(1.60)	−0.08	(0.05)	−1.556	0.120
Employment status						
Employed^*^						
Unemployed	3.35	(2.11)	0.08	(0.05)	1.589	0.112
Retired	6.67	(2.59)	0.18	(0.07)	2.576	0.010
Others	−1.71	(2.08)	−0.04	(0.05)	−0.821	0.412
Chinese medicine specialty visited						
Herbal medicine^*^						
Bone setting/massage	0.41	(2.48)	0.01	(0.04)	0.165	0.869
Acupuncture	1.58	(2.01)	0.04	(0.04)	0.785	0.432
Reason for consultation						
Episodic condition^*^						
Chronic condition	−1.78	(1.41)	−0.06	(0.05)	−1.265	0.206
Both conditions/others	−3.27	(2.38)	−0.06	(0.05)	−1.375	0.169
Self perceived health status						
Excellent /very good^*^						
Good	1.05	(2.45)	0.03	(0.07)	0.429	0.668
Fair	0.68	(2.34)	0.02	(0.08)	0.290	0.772
Poor	5.37	(2.99)	0.11	(0.06)	1.794	0.073
Usage conventional specialist services in public sector						
No^*^						
Yes	−1.16	(1.39)	−0.04	(0.05)	−0.835	0.404

Keys: PCAT, Primary Care Assessment Tool; CCTRCM, Clinical Centers for Teaching & Research in Chinese Medicine; NGO, Non Governmental Organization.

^†^Adjusted coefficients and standard errors derived from multiple linear regression using full information maximum likelihood.

^*^Reference group.

**Table 4:** Association between PCAT Total Score and Patients’ Characteristics in CCTRCM: Multiple Linear Regression.

	Unstandardized β_adj_ (SE)^†^	Standardized β_adj_ (SE)^†^	Z	*p*
Gender				
Male^*^				
Female	−1.27 (1.83)	−0.05 (0.08)	−0.690	0.490
Age				
15–29^*^				
30–39	5.02 (3.80)	0.13 (0.10)	1.322	0.186
40–49	2.84 (4.19)	0.10 (0.15)	0.678	0.498
50–59	7.62 (4.22)	0.28 (0.15)	1.806	0.071
60–69	5.78 (5.15)	0.20 (0.18)	1.123	0.262
>70	5.54 (6.30)	0.14 (0.15)	0.880	0.379
Type of housing				
Private housing^*^				
Public housing/others	0.60 (1.92)	0.03 (0.08)	0.313	0.755
Marital status				
Married/cohabitation^*^				
Single	3.02 (2.99)	0.12 (0.12)	1.008	0.313
Divorced/separated/widowed/others	1.89 (3.30)	0.05 (0.08)	0.574	0.566
Education level				
Tertiary education or above^*^				
Primary education or below	−7.32 (3.34)	−0.24 (0.11)	−2.192	0.028
Secondary education	−3.21 (2.11)	−0.14 (0.09)	−1.523	0.128
Employment status				
Employed^*^				
Unemployed	7.41 (2.79)	0.22 (0.08)	2.654	0.008
Retired	6.11 (3.65)	0.19 (0.11)	1.673	0.094
Others	5.96 (3.24)	0.15 (0.08)	1.842	0.065
Need to pay for consultation fee				
No^*^				
Yes	3.51 (2.19)	0.14 (0.09)	1.601	0.109
Chinese medicine specialty visited				
Herbal medicine^*^				
Bone setting/massage	−2.55 (2.72)	−0.07 (0.08)	−0.938	0.348
Acupuncture	−8.39 (2.89)	−0.22 (0.08)	−2.899	0.004
Reason for consultation				
Episodic illness^*^				
Chronic condition	−1.06 (1.95)	−0.05 (0.08)	−0.544	0.587
Both conditions/others	−1.40 (3.02)	−0.04 (0.08)	−0.462	0.644
Self perceived health status				
Excellent /very good^*^				
Good	−2.24 (3.00)	−0.09 (0.12)	−0.747	0.455
Fair	−3.16 (3.09)	−0.14 (0.13)	−1.022	0.307
Poor	−2.81 (4.10)	−0.07 (0.10)	−0.685	0.493
Usage conventional specialist services in public sector				
No^*^				
Yes	1.85 (2.20)	0.08 (0.09)	0.843	0.399

Key: PCAT, Primary Care Assessment Tool; CCTRCM, Clinical Centers for Teaching & Research in Chinese Medicine.

^†^Adjusted coefficients and standard errors derived from multiple linear regression using full information maximum likelihood.

^*^Reference group.
